# Perspective: Re-defining “Pheromone” in a Mammalian Context to Encompass Seminal Fluid

**DOI:** 10.3389/fvets.2021.819246

**Published:** 2022-01-20

**Authors:** Sarah A. Robertson, Graeme B. Martin

**Affiliations:** ^1^The Robinson Research Institute, Adelaide Medical School, University of Adelaide, Adelaide, SA, Australia; ^2^UWA School of Agriculture and Environment, UWA Institute of Agriculture, University of Western Australia, Crawley, WA, Australia

**Keywords:** pheromone, gonadotrophins, seminal fluid, immune response, hypothalamic-pituitary axis, uterus, cryptic female choice

## Abstract

The classical view of “pheromone”—an air-borne chemical signal—is challenged by the camelids in which ovulation is triggered by ß-nerve growth factor carried in seminal plasma, effectively extending the pheromone concept to a new medium. We propose further extension of “pheromone” to include a separate class of seminal fluid molecules that acts on the female reproductive tract to enhance the prospect of pregnancy. These molecules include transforming growth factor-ß, 19-OH prostaglandins, various ligands of Toll-like receptor-4 (TLR4), and cyclic ADP ribose hydrolase (CD38). They modulate the immune response to “foreign” male-derived histocompatibility antigens on both sperm and the conceptus, determine pre-implantation embryo development, and then promote implantation by increasing uterine receptivity to the embryo. The relative abundance of these immunological molecules in seminal plasma determines the strength and quality of the immune tolerance that is generated in the female. This phenomenon has profound implications in reproductive biology because it provides a pathway, independent of the fertilizing sperm, by which paternal factors can influence the likelihood of reproductive success, as well as the phenotype and health status of offspring. Moreover, the female actively participates in this exchange—information in seminal fluid is subject to “cryptic female choice,” a process by which females interrogate the reproductive fitness of prospective mates and invest reproductive resources accordingly. These processes participate in driving the evolution of male accessory glands, ensuring optimal female reproductive investment and maximal progeny fitness. An expanded pheromone concept will avoid a constraint in our understanding of mammalian reproductive biology.

## Introduction

While the theme of this special issue of *Frontiers in Veterinary Science* is focused clearly on signals transferred from males to females in semen, we would like to add an extra dimension to the discussion—the concept of “pheromone.” The original approach to male-female chemical signaling emerged from the field of entomology, for which pheromones were defined by Karlson and Lüscher in 1959 (1): “… substances which are secreted to the outside by an individual and received by a second individual of the same species, in which they release a specific reaction, for example, a definite behavior or a developmental process.”

A case for relaxing the definition to accommodate the “ram effect” in sheep was originally made in 1986 (2) and subsequently updated in 2012 (3). In this paper, we will use the “ram effect” as a primary example of a pheromone that has profound effects on mammalian reproduction (review: 2). It involves an olfactory signal from *novel* rams that activates the hypothalamic-pituitary-gonadal (HPG) axis of anovulatory ewes, leading to ovulation within a few days. The phenomenon has been studied in depth (review: 3) and we now know:

1. The chemical information is transmitted primarily by the main olfactory system, with the accessory olfactory system playing a relatively minor role; the information is delivered to neuronal networks in the preoptic-hypothalamic continuum (including the KNDy cells) (4) that control the tonic, pulsatile secretion of GnRH/LH;
2. Fos expression studies have shown that, in the first 2 h of male exposure, cells are activated in the arcuate nucleus (ARC), the ventromedial nucleus of the hypothalamus (VMH) and the organum vasculosum of the lamina terminalis (OVLT); by 6 h after male exposure, cells in the preoptic area (POA) are also activated (5);
3. Fos expression studies have also shown that removal of males quickly decreases cell activity in the ARC, VMH and OVLT, but has a relatively small effect on POA cells, perhaps explaining the incomplete decline in the tonic GnRH/LH response (5);
4. The stimulus evokes a rapid increase in cell proliferation in the dentate gyrus of the hippocampus; the roles played by this site, as well as the cortical amygdala (6), are coherent with the importance of “olfactory memory” in the “ram effect” (review: 3);
5. The need for a pheromonal signature for each individual male, so they can be identified as either novel or familiar, implies a complex mixture of compounds in the chemical signal; at best, this chemistry is only partially characterized (review: 3);
6. The responses are learned by the ewe, and depend on sexual experience (review: 3);
7. Non-olfactory (auditory, visual) ram stimuli help to achieve the optimum response, but are unable to substitute for the full complement of sociosexual stimuli from a living ram (review: 3);
8. The neuroendocrine response and the proportion of ewes that subsequently ovulate vary within and between genotypes (review: 3);
9. Species specificity appears to be flexible—for example, odor from male goats can elicit the neuroendocrine response in ewes (review: 3).

In 2010, Wyatt (7) also expanded the pheromone concept to encompass more complex signals, a broader variety of physiological processes, and a greater number of species: “… molecules that are evolved signals, in defined ratios in the case of multiple component pheromones, which are emitted by an individual and received by a second individual of the same species, in which they cause a specific reaction, for example, a stereotyped behavior or a developmental process.” Wyatt (7) also defined “signature mixture” as a: “… variable chemical mixture (a subset of the molecules in an animal's chemical profile) learned by other conspecifics and used to recognize an animal as an individual ….”

Clearly, the “ram effect” in sheep fulfills both of these conditions. The question we pose here is: can we stretch the pheromone definition beyond olfactory signals to include semen, the complex biological and chemical mixture that is transmitted from males to females at mating?

## Induction of Ovulation by Seminal Fluid

Seminal fluid had long been seen as a passive carrier of spermatozoa, but that view is challenged fundamentally by the camelids, where exposure to seminal plasma at mating triggers ovulation (8–10). It is now known that this phenomenon is mediated by ß-nerve growth factor (NGF-β) that, through an as-yet unresolved pathway, elicits an ovulatory surge of LH, perhaps though action on GnRH neurons at the level of the median eminence (11–17). Induction of LH secretion by seminal plasma NGF-β is also thought to enhance the development and function of the corpus luteum in camelids (18).

The conceptual overlap of this phenomenon with the ram effect is self-evident—the major difference being air-borne vs. semen-borne chemical signals. Our view is that NGF-β in seminal plasma is indeed a pheromone and, moreover, we need to expand this discussion even further to encompass molecular components of seminal fluid that do not directly affect the HPG axis.

## Effects of Seminal Fluid on the Female Reproductive Tract

A substantial body of work over the last decade or so has clearly demonstrated the profound effects of seminal fluid—the seminal plasma as well as agents carried on the surface of sperm—on the reproductive tract and physiology of recipient females in several mammalian species, including humans, mice, pigs, dogs, cattle and sheep (19–21). Seminal fluid is produced mostly in the seminal vesicles and is rich in inorganic salts and micronutrients (e.g., potassium, zinc), carbohydrates (particularly reducing sugars such as fructose), lipid derivatives (including prostaglandins, phosphorylcholine), extracellular vesicles, and an array of glycoproteins including lactoferrin, proteinase inhibitors and cytokines (20). It has long been known that these factors play functional roles in fertilization, including semen coagulation, sperm motility and capacitation. Now, it is clear that they also elicit direct paracrine and endocrine responses that induce changes in gene expression leading to an immune response in the cervix, uterus, oviduct, and ovary of the female tract, with substantial consequences for the success of conception, embryo implantation and pregnancy (22, 23). In turn, as with the invertebrates, these processes provide pathways (independent of the sperm) by which paternal factors can influence the phenotype and health status of offspring after birth (24, 25). In particular, by modulating the immune response of the female receiving “foreign” male-derived histocompatibility antigens (or transplantation proteins), they determine pre-implantation embryo development and then promote implantation by increasing receptivity to the embryo (19, 26).

The signals known to be responsible for the immunological effects are the isoforms of transforming growth factor-ß (TGFß), TGFß1, TGFß2 and TGFß3, 19-OH prostaglandins (19-OH PGE), various ligands of Toll-like receptor-4 (TLR4), and cyclic ADP ribose hydrolase (CD38) (21, 27, 28). These molecules bind to epithelial cells lining the female reproductive tract where they elicit a surge in cytokine expression that induces a rapid recruitment of immune cells into the local endometrial and cervical tissue. Ultimately, the female immune response is “primed” to seminal fluid antigens and tolerance-conferring regulatory T-cells are activated to permit the presence on the conceptus of paternally-inherited histocompatibility antigens (29). Consequently, the generation of effector immunity against sperm antigens is suppressed and the female immune system is induced to tolerate the histocompatibility antigens. These same antigens are present on embryos produced by the male gametes, so the immune response to seminal fluid effectively “primes” the female immune response to facilitate survival of embryos sired by that male (26, 29).

These immunological effects of seminal fluid have been reported for a wide variety of mammalian species. For example, in goats and sheep, mating induces a transient inflammatory response with hallmark recruitment of macrophages and neutrophils into the cervical and uterine tissues (19, 30, 31). In common with all other species studied to date, the key active signaling factor in goat and sheep seminal plasma is TGFβ (32). Analysis of the uterine inflammatory response after mating in sheep demonstrates that, as in mice (23, 24), both sperm and seminal fluid contribute to infiltration by macrophages and neutrophils, typically leading to induction of seminal fluid-mediated IL8 and GM-CSF secretion (33).

Seminal fluid also influences the expression of the cytokines and growth factors that modulate embryo survival and developmental programming (34). Several cytokines released by the oviduct and uterine epithelium exert permissive or inhibitory effects on embryo survival and development. Growth factors that affect embryo development are induced in the uterus and oviduct of mice, pigs, sheep, and other species where seminal fluid factors access the uterus and oviduct (24, 28, 33, 35, 36). When oviductal cytokine expression is disrupted in early pregnancy by mating females with males rendered deficient in seminal plasma, fewer embryos survive and those that do are less able to implant and develop (24).

In addition to the immunological effects and the ovulatory response, seminal fluid affects the ovary where as-yet unidentified components facilitate luteal development and progesterone secretion (37), after being delivered by unique counter-current exchange mechanisms where small molecules are transferred to the ovarian artery from the uterine vein (38). One candidate, relaxin, is known to be carried by seminal plasma in rodents (39) and has been shown to promote ovulation through connective tissue remodeling in the follicle wall (40).

## Conclusion

Clearly, seminal fluid carries molecular agents that act in the female reproductive tract to alter female physiology in a most fundamental way to promote reproductive success, and these effects go well beyond the induction of ovulation. We contend that the chemical components of seminal fluid meet the definition of “pheromone”—they act outside the body of the originating male to affect not only the HPG axis of the female but to change her immune system and thus enhance the prospect of a successful pregnancy.

Interestingly, this perspective brings us back to the world of invertebrates where the role of seminal fluid in regulating female reproductive physiology and behavior is also relevant (41–43). For instance, in *Drosophila*, male factors induce synthesis of antimicrobial protein and influence brain function to reduce female receptivity to other males (44), and the seminal plasma protein, ovulin, stimulates octopamine neuronal signaling and induces ovulation (45), reminiscent of the induction of ovulation by seminal fluid NGF-ß in camelids.

These phenomena have profound implications for reproductive biology because the capacity of the male to induce a response in the female will determine the success of the transmission of the male germ line. Moreover, the female becomes an active participant in this exchange—information provided in seminal fluid is subject to “cryptic female choice,” a process by which females interrogate the reproductive fitness of prospective male partners and invest reproductive resources accordingly (46). Resources invested in the promotion of successful fertilization contribute to the evolution of male accessory glands (47), helping to ensure optimal female reproductive investment and maximal progeny fitness (48).

By expanding the pheromone concept, we avoid being constrained in our understanding of the mammalian reproductive biology, including that of humans.

## Author Contributions

All authors listed have made a substantial, direct, and intellectual contribution to the work and approved it for publication.

## Conflict of Interest

The authors declare that the research was conducted in the absence of any commercial or financial relationships that could be construed as a potential conflict of interest.

## Publisher's Note

All claims expressed in this article are solely those of the authors and do not necessarily represent those of their affiliated organizations, or those of the publisher, the editors and the reviewers. Any product that may be evaluated in this article, or claim that may be made by its manufacturer, is not guaranteed or endorsed by the publisher.

## References

[1] Karlson P Lüscher M. Pheromones: a new term for a class of biologically active substances. Nature. (1959) 183:55–6. 10.1038/183055a0 13622694

[2] Martin GB Oldham CM Cognié Y Pearce DT. The physiological responses of anovulatory ewes to the introduction of rams—a review. Livestock Prod Sci. (1986) 15:219–47. 10.1016/0301-6226(86)90031-X

[3] Hawken PAR Martin GB. Socio-sexual stimuli and GnRH/LH secretion in sheep and goats. Domest Anim Endocrinol. (2012) 43:85–94. 10.1016/j.domaniend.2012.03.005 22533940

[4] Fabre-Nys C Cognié J Dufourny L Ghenim M Martinet S Lasserre O. The two populations of kisspeptin neurons are involved in the ram induced LH pulsatile secretion and LH surge in anestrous ewes. Endocrinology. (2017) 158:3914–28. 10.1210/en.2017-00429 28938486

[5] Hawken PAR Smith JT Jorre de St Jorre T Esmaili T Scott CJ Rodger J. Patterns of preoptic–hypothalamic neuronal activation and LH secretion in female sheep following the introduction and withdrawal of novel males. Reprod Fertil Develop. (2019) 31:1674–81. 10.1071/RD19079 31511142

[6] Gelez H Archer E Chesneau D Magallon T Fabre-Nys C. Inactivation of the olfactory amygdala prevents endocrine response to male odour in anestrus ewes. Eur J Neurosci. (2004) 19:1581–90. 10.1111/j.1460-9568.2004.03261.x 15066154

[7] Wyatt TD. Pheromones and signature mixtures: defining species-wide signals and variable cues for identity in both invertebrates and vertebrates. J Comp Physiol. (2010) 196:685–700. 10.1007/s00359-010-0564-y 20680632

[8] Fernandez-Baca S Madden DH Novoa C. Effect of different mating stimuli on induction of ovulation in the alpaca. J Reprod Fertil. (1970) 22:261–7. 10.1530/jrf.0.0220261 5464117

[9] Chen BX Yuen ZX Pan GW. Semen-induced ovulation in the bactrian camel (*Camelus bactrianus*). J Reprod Fertil. (1985) 74:335–9. 10.1530/jrf.0.0740335 3900379

[10] Adams GP Ratto MH Huanca W Singh J. Ovulation-inducing factor in the seminal plasma of alpacas and llamas. Biol Reprod. (2005) 73:452–7. 10.1095/biolreprod.105.040097 15888733

[11] Ratto MH Huanca W Adams GP. Ovulation-inducing factor: a protein component of llama seminal plasma. BMC Reprod Biol Endocrinol. (2010) 8:44. 10.1186/1477-7827-8-44 20462434 PMC2881935

[12] Ratto MH Leduc YA Valderrama XP van Straaten KE Delbaere LTJ Pierson RA. The nerve of ovulation inducing factor in semen. Proc Natl Acad Sci USA. (2012) 109:15042–7. 10.1073/pnas.1206273109 22908303 PMC3443178

[13] Kershaw-Young CM Druart X Vaughan J Maxwell WMC. ß-Nerve growth factor is a major component of alpaca seminal plasma and induces ovulation in female alpacas. Reprod Fertil Develop. (2012) 24:1093–97. 10.1071/RD12039 22951217

[14] Stuart CC Vaughan JL Kershaw-Young CM Wilkinson J Bathgate R de Graaf SP. Effects of varying doses of ß-nerve growth factor on the timing of ovulation, plasma progesterone concentration and corpus luteum size in female alpacas (*Vicugna pacos*). Reprod Fertil Develop. (2014) 27:1181–6. 10.1071/RD14037 24965784

[15] Carrasco RA Pezo S Adams GP. Evidence for the LH-releasing pathway of seminal plasma NGF in male camelids. Theriogenology. (2021) 164:100–4. 10.1016/j.theriogenology.2021.01.014 33582512

[16] Carrasco RA Leonardi CE Pezo S Adams GP. Hypothalamic involvement and the role of progesterone in the NGF-induced LH surge pathway. Reproduction. (2021) 162:171–9. 10.1530/REP-20-0647 34128825

[17] Carrasco RA Singh J Ratto HR Adams GP. Neuroanatomical basis of the nerve growth factor ovulation-induction pathway in llamas. Biol Reprod. (2020) 104:578–88. 10.1093/biolre/ioaa223 33331645

[18] Silva M Ulloa-Leal C Norambuena C Fernandez A Adams GP Ratto MH. Ovulation-inducing factor (OIF/NGF) from seminal plasma origin enhances corpus luteum function in llamas regardless the preovulatory follicle diameter. Anim Reprod Sci. (2014) 148:221–7. 10.1016/j.anireprosci.2014.05.012 24950997

[19] Rodríguez-Martínez H Kvist U Ernerudh J Sanz L Calvete JJ. Seminal plasma proteins: what role do they play? Am J Reprod Immunol. (2011) 66 (Suppl. 1):11–22. 10.1111/j.1600-0897.2011.01033.x 21726334

[20] McGraw LA Suarez SS Wolfner MF. On a matter of seminal importance. Bioessays. (2015) 37:142–7. 10.1002/bies.201400117 25379987 PMC4502980

[21] Schjenken JE Robertson SA. Seminal fluid and immune adaptation for pregnancy—comparative biology in mammalian species. Reprod Domest Anim. (2014) 49 (Suppl 3):27–36. 10.1111/rda.12383 25220746

[22] Schjenken JE Robertson SA. The female response to seminal fluid. Physiol Rev. (2020) 100:1077–117. 10.1152/physrev.00013.2018 31999507

[23] Schjenken JE Sharkey DJ Green ES Chan HY Matias RA Moldenhauer LM. Sperm modulate uterine immune parameters relevant to embryo implantation and reproductive success in mice. Commun Biol. (2021) 4:572. 10.1038/s42003-021-02038-9 33990675 PMC8121928

[24] Bromfield JJ Schjenken JE Chin PY Care AS Jasper MJ Robertson SA. Maternal tract factors contribute to paternal seminal fluid impact on metabolic phenotype in offspring. Proc Natl Acad Sci USA. (2014) 111:2200–05. 10.1073/pnas.1305609111 24469827 PMC3926084

[25] Lane M Robker RL Robertson SA. Parenting from before conception. Science. (2014) 345:756–60. 10.1126/science.1254400 25124428

[26] Robertson SA Care AS Moldenhauer LM. Regulatory T cells in embryo implantation and the immune response to pregnancy. J Clin Invest. (2018) 128:4224–35. 10.1172/JCI122182 30272581 PMC6159994

[27] Kim BJ Choi YM Rah SY Park DR Park SA Chung YJ. Seminal CD38 is a pivotal regulator for fetomaternal tolerance. Proc Natl Acad Sci USA. (2015) 112:1559–64. 10.1073/pnas.1413493112 25591581 PMC4321276

[28] Schjenken JE Glynn DJ Sharkey DJ Robertson SA. TLR4 signaling is a major mediator of the female tract response to seminal fluid in mice. Biol Reprod. (2015) 93:68. 10.1095/biolreprod.114.125740 26157066

[29] Robertson SA Guerin LR Bromfield JJ Branson KM Ahlstrom AC Care AS. Seminal fluid drives expansion of the CD4^+^CD25^+^ T regulatory cell pool and induces tolerance to paternal alloantigens in mice. Biol Reprod. (2009) 80:1036–45. 10.1095/biolreprod.108.074658 19164169 PMC2849830

[30] Mattner PE. The distribution of spermatozoa and leucocytes in the female genital tract in goats and cattle. J Reprod Fertil. (1968) 17:253–61. 10.1530/jrf.0.0170253 5749378

[31] Scott JL Ketheesan N Summers PM. Leucocyte population changes in the reproductive tract of the ewe in response to insemination. Reprod Fertil Dev. (2006) 18:627–34. 10.1071/rd05165 16930509

[32] Das A Uddin AM Uddin MB Rahman A Hossain MK Atikuzzaman M. Seminal plasma contains TGF-ß and CXCL10 but sperm washing before cryopreservation is beneficial for post-thawing sperm motility in Black Bengal goats (*Capra hircus*). Am J Reprod Immunol. (2020) 84:e13301. 10.1111/aji.13301 32659038

[33] Scott JL Ketheesan N Summers PM. Spermatozoa and seminal plasma induce a greater inflammatory response in the ovine uterus at oestrus than dioestrus. Reprod Fertil Dev. (2009) 21:817–26. 10.1071/RD09012 19698286

[34] Robertson SA Chin PY Glynn DJ Thompson JG. Peri-conceptual cytokines—setting the trajectory for embryo implantation, pregnancy and beyond. Am J Reprod Immunol. (2011) 66(Suppl 1):2–10. 10.1111/j.1600-0897.2011.01039.x 21726333

[35] Robertson SA Mayrhofer G Seamark RF. Uterine epithelial cells synthesize granulocyte-macrophage colony-stimulating factor and interleukin-6 in pregnant and nonpregnant mice. Biol Reprod. (1992) 46:1069–79. 10.1095/biolreprod46.6.1069 1391304

[36] O'Leary S Jasper MJ Warnes GM Armstrong DT Robertson SA. Seminal plasma regulates endometrial cytokine expression, leukocyte recruitment and embryo development in the pig. Reprod. (2004) 128:237–47. 10.1530/rep.1.00160 15280563

[37] O'Leary S Jasper MJ Robertson SA Armstrong DT. Seminal plasma regulates ovarian progesterone production, leukocyte recruitment and follicular cell responses in the pig. Reprod. (2006) 132:147–58. 10.1530/rep.1.01119 16816340

[38] Krzymowski T Stefanczyk-Krzymowska S Koziorowski M. Counter current transfer of PGF2 alpha in the mesometrial vessels as a mechanism for prevention of luteal regression in early pregnancy. Acta Physiol Pol. (1989) 40:23–34. 2690571

[39] Glynn DJ Heng K Russell DL Sharkey DJ Robertson SA Anand-Ivell R. Male seminal relaxin contributes to induction of the post-mating cytokine response in the female mouse uterus. Front Physiol. (2017) 8:422. 10.3389/fphys.2017.00422 28674503 PMC5474474

[40] Brännström M MacLennan AH. Relaxin induces ovulations in the *in-vitro* perfused rat ovary. Hum Reprod. (1993) 8:1011–14. 10.1093/oxfordjournals.humrep.a138184 8408479

[41] Gillott C. Male accessory gland secretions: modulators of female reproductive physiology and behavior. Annu Rev Entomol. (2003) 48:163–84. 10.1146/annurev.ento.48.091801.112657 12208817

[42] Avila FW Sirot LK LaFlamme BA Rubinstein CD Wolfner MF. Insect seminal fluid proteins: identification and function. Annu Rev Entomol. (2011) 56:21–40. 10.1146/annurev-ento-120709-144823 20868282 PMC3925971

[43] Simmons LW Lovegrove M. Nongenetic paternal effects via seminal fluid. Evol Lett. (2019) 3:403–11. 10.1002/evl3.124 31388449 PMC6675144

[44] Ram KR Wolfner MF. Sustained post-mating response in Drosophila melanogaster requires multiple seminal fluid proteins. PLoS Genet. (2007) 3:e238. 10.1371/journal.pgen.0030238 18085830 PMC2134937

[45] Rubinstein CD Wolfner MF. Drosophila seminal protein ovulin mediates ovulation through female octopamine neuronal signaling. Proc Natl Acad Sci USA. (2013) 110:17420–25. 10.1073/pnas.1220018110 24101486 PMC3808635

[46] Roldan ER Gomendio M Vitullo AD. The evolution of eutherian spermatozoa and underlying selective forces: female selection and sperm competition. Biol Rev Camb Philos Soc. (1992) 67:551–93. 10.1111/j.1469-185x.1992.tb01193.x 1463811

[47] Karn RC Clark NL Nguyen ED Swanson WJ. Adaptive evolution in rodent seminal vesicle secretion proteins. Mol Biol Evol. (2008) 25:2301–10. 10.1093/molbev/msn182 18718917 PMC2727389

[48] Eberhard WG. Postcopulatory sexual selection: Darwin's omission and its consequences. Proc Natl Acad Sci USA. (2009) 106(Suppl 1):10025–32. 10.1073/pnas.0901217106 19528642 PMC2702800

